# De Novo Variant in *GBX1* Gene Associated With Developmental Delay and Focal Epilepsy

**DOI:** 10.1002/mgg3.70114

**Published:** 2025-06-16

**Authors:** Bingbing Zhang, Xiaohua Li, Xiao Qian, Jihong Tang

**Affiliations:** ^1^ Children's Hospital of Soochow University Jiangsu Province China; ^2^ The First People's Hospital of Lianyungang Jiangsu Province China; ^3^ Cipher Gene LLC Beijing China

**Keywords:** developmental delay, focal epilepsy, GBX1, whole exome sequencing

## Abstract

**Background:**

The gastrulation brain homeobox (Gbx) family, including GBX1 and GBX2, is crucial for hindbrain development and contributes to the morphogenesis of the midbrain–hindbrain boundary (MHB). While the role of the *GBX1* gene in the development of the human nervous system remains to be elucidated, its variant in humans has not previously been reported to be associated with disease.

**Methods:**

The patient presenting with sleep panic attacks underwent comprehensive clinical assessments, including electroencephalograph (EEG), magnetic resonance imaging (MRI), and genetic testing through whole exome sequencing (WES). Zebrafish models were generated through *gbx1* gene crispants to investigate the functional impact of identified genetic variants.

**Results:**

The patient in our study was diagnosed with focal epilepsy through long‐range EEG. WES revealed a de novo *GBX1* gene variant [NM_001098834.3: c.910C>T (p.Gln304*)]. In zebrafish larvae with *gbx1* gene disruption, significant abnormalities were observed in the morphology of the interocular area. Furthermore, these larvae exhibited an increased susceptibility to neurophysiological abnormalities associated with epileptiform activity.

**Conclusion:**

Our study is the first to identify an association between the *GBX1* gene variant and focal epilepsy. The zebrafish models confirmed the presence of related phenotypes in the *gbx1*‐Cas9. These findings underscore the significance of the *GBX1* gene in neurological function.

## Graphical abstract

We first identified an association between the *GBX1* gene variant and focal epilepsy, and observed related phenotypes in the *gbx1*‐Cas9 zebrafish model.

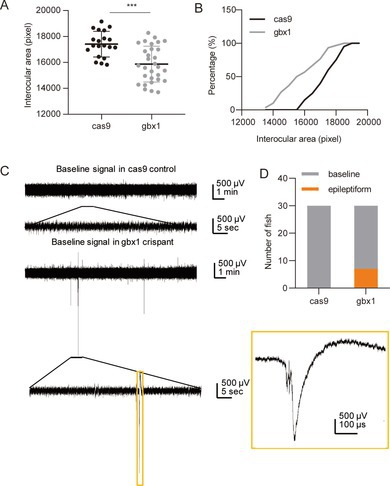

## 1 Introduction

Epilepsy is a neurological disorder associated with persistent disturbances in brain function that can lead to recurrent seizures (Fisher et al. 2014). The classification of epilepsy is typically based on the origin of the seizure, with the condition being categorized as either focal or generalized. Focal epilepsy of unknown etiology constitutes the most prevalent category of patients diagnosed with epilepsy for the first time (Zarrelli et al. 1999). About 25% of patients with focal epilepsy have no clear cause (Jobst et al. 2000). Recent advances in genomic technology, especially the application of whole exome sequencing (Westerfield 2000), have provided new tools and methods for investigating epilepsy etiology (Dunn et al. 2018). So far, the pathway most frequently addressed in the literature is the mechanistic target of the rapamycin complex 1 (mTORC1) pathway. DEPDC5, NPRL2, and NPRL3 are three subunits of the GATOR1 complex that regulate mTORC1 signaling. Mutations in the genes encoding these subunits have been identified in a number of patients and families with focal epilepsy. Of these, *DEPDC5* mutations appear to be the most prevalent, accounting for approximately 10% of familial focal epilepsy cases in different cohorts (Ricos et al. 2016; Bisulli et al. 2016; Dibbens et al. 2013). In addition to the mTORC1 pathway, other genetic causes of focal epilepsy include mutations in the *CHRNA2*, *CHRNA4*, *CHRNB2*, *KCNT1*, and *LGI1* genes (Steinlein et al. 2012; Heron et al. 2012; McTague et al. 2018). Mutations in these genes further reveal the complex molecular mechanisms of focal epilepsy by affecting ion channel function, neural network stability, or cell signaling.

The gastrulation brain homeobox (Gbx) family plays a crucial role in hindbrain development (Zhao et al. 2024). Comprising two genes, *GBX1* and *GBX2*, these genes encode homeodomain‐containing transcription factors that are evolutionarily conserved across various organisms (Rhinn et al. 2003). In zebrafish, the gbx1 gene exhibits dynamic expression patterns during hindbrain development. At 80% of epiboly, it spans the hindbrain primordium and contributes to the morphogenesis of the midbrain–hindbrain boundary (MHB) (Rhinn et al. 2009). Overexpression of either gbx1 or gbx2 suppresses forebrain and midbrain formation while promoting hindbrain development (Kikuta et al. 2003). Currently, the *GBX1* gene has not been reported to be associated with diseases. However, according to previous experimental studies, the *GBX1* gene is crucial to the development of the nervous system.

Here, we report a Chinese girl who presented with developmental delays and experienced sudden awakenings with a fearful gaze, tense limbs, and twisting or circling movements. The focal epilepsy was confirmed through long‐range EEG testing. WES revealed a de novo truncated variant in the *GBX1* gene (c.910C>T, p.Gln304*). The gbx1‐cas9 zebrafish exhibited a significant reduction in the interocular area and a substantial increase in the occurrence of epileptiform activity compared to the Cas9 control group. Our finding of a *GBX1* gene variant is novel; we first identified a *GBX1* variant associated with developmental delay and focal epilepsy. Moreover, a correlation is demonstrated between the *GBX1* mutation and the occurrence of epilepsy in the zebrafish model.

## 2 Methods

### 2.1 Patients

A patient with sleep panic attacks was confirmed at Children's Hospital of Soochow University. Informed consent was obtained from the family following institutional guidelines. Ethics approval was granted by the human ethics committee of Children's Hospital of Soochow University. The patient underwent comprehensive clinical evaluations, including EEG and MRI performed with a 3 Tesla scanner. Laboratory tests and genetic tests were also conducted.

### 2.2 WES

Genomic DNA was extracted from peripheral blood samples of the patient and her parents. For the purpose of exome sequencing, the extracted DNA was fragmented, and libraries were captured using the IDT XGen Exome Research Panel. Paired‐end read sequences were generated on the NovaSeq 6000, achieving an average sequencing depth of over 100× and a 20× coverage greater than 98% for quality control standards. Sequences were aligned to the human reference genome GRCh38/hg38 utilizing the Burrows‐Wheeler Aligner (BWA) (Abuín et al. 2015), Samtools, and Picard software. The GATK software facilitated re‐alignment, duplicate sequence removal, and variant detection. The variants were annotated using Annovar software (Wang et al. 2010), and their pathogenicity was predicted with tools such as Mutation Taster, PolyPhen‐2, and SIFT. The variants were filtered based on minor allele frequency (MAF), inheritance patterns, clinical phenotype, and public databases. The pathogenicity of the candidate variants was assessed in accordance with the guidelines of the American College of Medical Genetics and Genomics (ACMG). Chromosome analysis, including evaluation for ring chromosomes, array comparative genomic hybridization (array CGH), and basal metabolic tests, was not conducted on the patient.

### 2.3 Zebrafish Maintenance

Adult zebrafish were kept in tanks with circulating water maintained at 28°C, following a 14/10‐h light/dark cycle, and fed twice daily (Z‐A‐D5 housing system, Haisheng, Shanghai). Embryos were obtained by standard mating methods (Westerfield 2000), and larvae were raised in embryo media containing 0.03% Instant Ocean and 0.0002% methylene blue (YHSZ‐003, Yahua, Shandong) in reverse osmosis‐distilled water. All procedures adhered to the Guide for the Care and Use of Animals (National Research Council Committee for the Update of the Guide for the, C. and A. Use of Laboratory 2011) and the guidelines established by Cipher Gene LLC.

### 2.4 Gene Editing and TIDE Assessment

The zebrafish genome includes the *GBX1* ortholog, *gbx1* (ENSDARG00000071418), which was identified using the DIOPT Ortholog Finder (Hu et al. 2011), showing a 64% protein identity with human GBX1. Single guide RNA (sgRNA) targets were selected via the CHOPCHOP online tool (Chang et al. 2023) and procured from GenScript. Two sgRNAs were designed (PAM sequence in lowercase): GGCCGCGTGCAGTGACCGGGAgg and CAGGACGCGTTACCGCCGGGcgg. To induce mutations in the target gene, approximately 2 nL of CRISPR complexes, containing sgRNAs (100 ng/μL each) and Cas9 protein (250 ng/μL), were injected into fertilized embryos at the 1–2 cell stage. At 24 h post‐injection, a subset of embryos was pooled for Sanger sequencing to verify mutagenesis efficacy using the TIDE (Tracking of Indels by DEcomposition) online tool (Brinkman et al. 2014). Subsequent genotyping was performed after phenotypic studies, with individual larvae sequenced and analysed via TIDE to confirm mutagenesis. Larvae with a TIDE efficacy below 5% were excluded from the phenotypic data analysis.

### 2.5 Imaging

At 4–5 days post‐fertilization (dpf), zebrafish larvae were positioned in a customized mini‐well plate (5 mm diameter and 1 mm depth) with one fish per well, oriented in the dorsal direction for both bright‐field and fluorescent imaging. The images were captured using a Touptek CCD camera (ToupTek, ToupCam) attached to a Nikon SMZ800N stereo microscope (Nikon Instruments Inc.), with 2× magnification for bright‐field and 4X magnification for larvae. The images were subsequently assessed for overall embryonic morphology and analysed in Fiji (ImageJ, National Institutes of Health) to measure eye distance, body length, and interocular area.

### 2.6 Electrophysiological Testing

Electrophysiological tests were conducted on zebrafish larvae at 5–6 dpf, divided into a Cas9 control group and a gbx1‐edited group. Individual larvae were placed in a sample dish and anesthetized with a drop of 300 μM pancuronium bromide E3 (P1918, Sigma‐Aldrich) solution for 3 min. Following anesthesia, larvae were positioned dorsal side up and immobilized using 2% low‐melting‐point agarose gel (A600015‐0025, Shenggong, Shanghai). Once the agarose solidified, the larvae were transferred to the experimental recording chamber, and E3 medium was added to cover the agarose and reference electrode. Local field potentials (LFP) were measured from the optic tectum using glass microelectrodes filled with 2 M sodium chloride (10,019,318, Hushi, Shanghai). The electrical signals were recorded using a high‐impedance amplifier (1700, A‐M Systems). Signals were digitized via an analog‐to‐digital converter (USB‐2231, Measurement Computing), with a sampling rate of 10 kHz and a filter setting of 1 Hz to 5 kHz. Each measurement lasted for 15 min. Data acquisition and analysis were performed using the open‐source software DClamp.

### 2.7 Statistics

Statistical analyses were performed with Prism 8 (GraphPad Software). An unpaired *t*‐test was used for two‐variable comparisons. Significance for all tests was defined as *p < 0.05; **p < 0.01; ***p < 0.001.

## 3 Results

### 3.1 Case Report

The patient is currently 9 years and 4 months old, and was born at full term with a birth weight of 2.25 kg. While prenatal assessments did not reveal any overt anomalies, a thin umbilical cord was noted at birth. The attainment of developmental milestones, including rolling over, sitting, walking, and speaking, was exhibited with a delay of 1–3 months compared to children of similar age. Commencing at 12 months of age, the patient has been undergoing ongoing rehabilitative interventions encompassing physical exercises, sensory integration techniques, and speech therapy. These therapeutic measures have been consistently administered up to the present time.

Since July 2019, the patient has experienced episodes of abrupt awakening during periods of sleep at night or napping, characterized by sudden awakening, turning over into a prone position, fearful facial expressions with a vacant gaze, increased muscle tone, intermittent hip‐twisting movements, or getting out of bed and walking in circles, and exhibiting partial responsiveness to calling. These episodes occur approximately 1–2 times per month, and the patient can return to sleep following a brief period of reassurance.

Since October 2019, the patient's upper respiratory tract infection led to an increased frequency of episodes, occurring every 2–3 days and lasting 12–13 min. Episodes usually accrued at 1–2 h after sleep at night, 5–7 am before awake, and 0.5–1 h after napping. Episodes of abrupt awakening with legs lightly shaking, breathing rapid, wide eyes, head and back sweating, hands and feet cold, and little response to calling. This could resolve after a few seconds or minutes.

**FIGURE 1:**
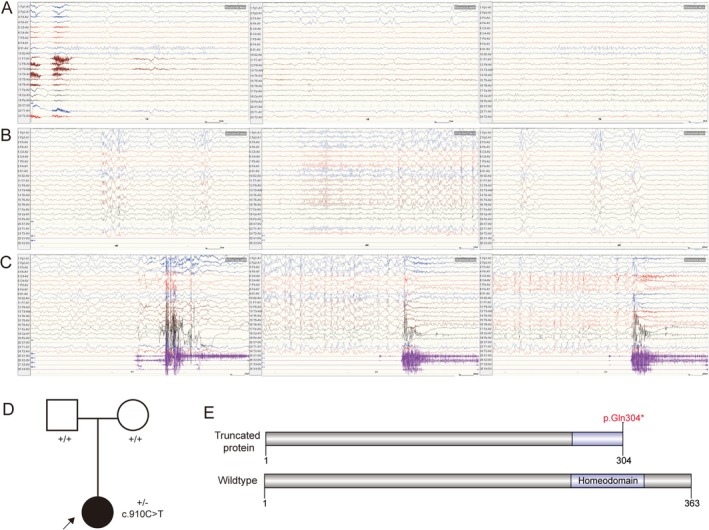
The clinical data and *GBX1* variant in the patient. A–C. Abnormal EEG. (A) Normal background rhythm with 8–10 Hz *α* waves in occipitals during wakefulness with eyes closed. High‐amplitude slow wave bursts in the right frontal region and sharp‐slow wave bursts in both occipitals. (B) During sleep, generalized spikes, spike‐slow waves, and polyspike bursts, especially in the left frontal region, with scattered bursts in the right frontal, occipital, and posterior temporal regions. (C) Focal seizures originating from the right frontal region, evolving from slow waves to fast‐wave activity. (D) The pedigree for the family. The patient was highlighted with an arrow with the filled symbol. (E) The schematic diagram for wildtype and p.Gln304* GBX1 protein.

The patient underwent a long‐range EEG examination on July 25, 2023, which resulted in a diagnosis of focal epilepsy. During periods of wakefulness, the background rhythm was normal with 8–10 Hz *α* waves in both occipitals. High‐amplitude slow waves were seen in bursts over the right frontal pole and right frontal midline. Sharp‐slow wave and spike‐slow wave bursts were present in both occipital regions (Figure 1A). During sleep, generalized spike, spike‐slow wave, and polyspike bursts were observed, most prominent over the left frontal region. High‐amplitude spikes and spike‐slow waves were seen in scattered bursts over the right frontal, right occipital, and right posterior temporal regions (Figure 1B). Several focal seizures originating from the right frontal region were recorded. The seizures manifested as low‐to‐medium amplitude slow waves in the right frontal region, which rapidly progressed into low‐amplitude fast‐wave activity, predominantly in the right frontal region (Figure 1C). The MRI (performed at the age of 4 years and 6 months), cranial nerves, limb muscle strength, and muscle tone were normal.

The patient was initiated on sodium valproate (VPA) in May 2022. The current dosage was VPA 6 mL twice a day, and adding clonazepam 0.25 tablets every night. The episodes decreased to 1–2 times a month, and the degree was slightly short. The symptoms changed to suddenly waking up after falling asleep at night or during a nap, turning over, and lying on her stomach. Her eyes were fearful, her hands and feet cold, her limbs tense, twisting her buttocks or turning in circles intermittently. She has no obvious response or a slight response to calling. She could wake up completely and return to normal after a few minutes to more than 10 min.

The consistent rehabilitation training starting from 1 year old showed limited effectiveness. She is 7 years old now. She is difficult to learn; she cannot remember knowledge from being repeatedly taught.

### 3.2 Identification of *GBX1* Gene Variant in Our Patient

In order to clarify the etiology of our patient, WES was performed using peripheral blood samples from the patient and her family. A de novo variant in the *GBX1* gene (NM_001098834.3: c.910C>T, p.Gln304*) was found in our patient (Figure 1D,E). It is rare and not found in public databases, including gnomAD (http://gnomad‐sg.org/, accession date: June 4, 2024), Exome Aggregation Consortium (https://ngdc.cncb.ac.cn/databasecommons/database/id/3774, June 4, 2024) and ClinVar (https://www.ncbi.nlm.nih.gov/clinvar/, accession date: June 4, 2024). The MutationTaster prediction result indicates that the variant is disease‐causing (Rhinn et al. 2003), suggesting that it may have a deleterious effect on gene function and be associated with disease. The variant p.Gln304* in the *GBX1* gene leads to a premature stop codon.

### 3.3 Zebrafish Verification

To investigate the function of gbx1 in the early embryonic development of zebrafish. A *gbx1* crispants model was successfully generated. Subsequently, an assessment was conducted of the phenotypic impact of *gbx1* disruption in zebrafish larvae through morphological, behavioral, and electrophysiological analyses.

**FIGURE 2:**
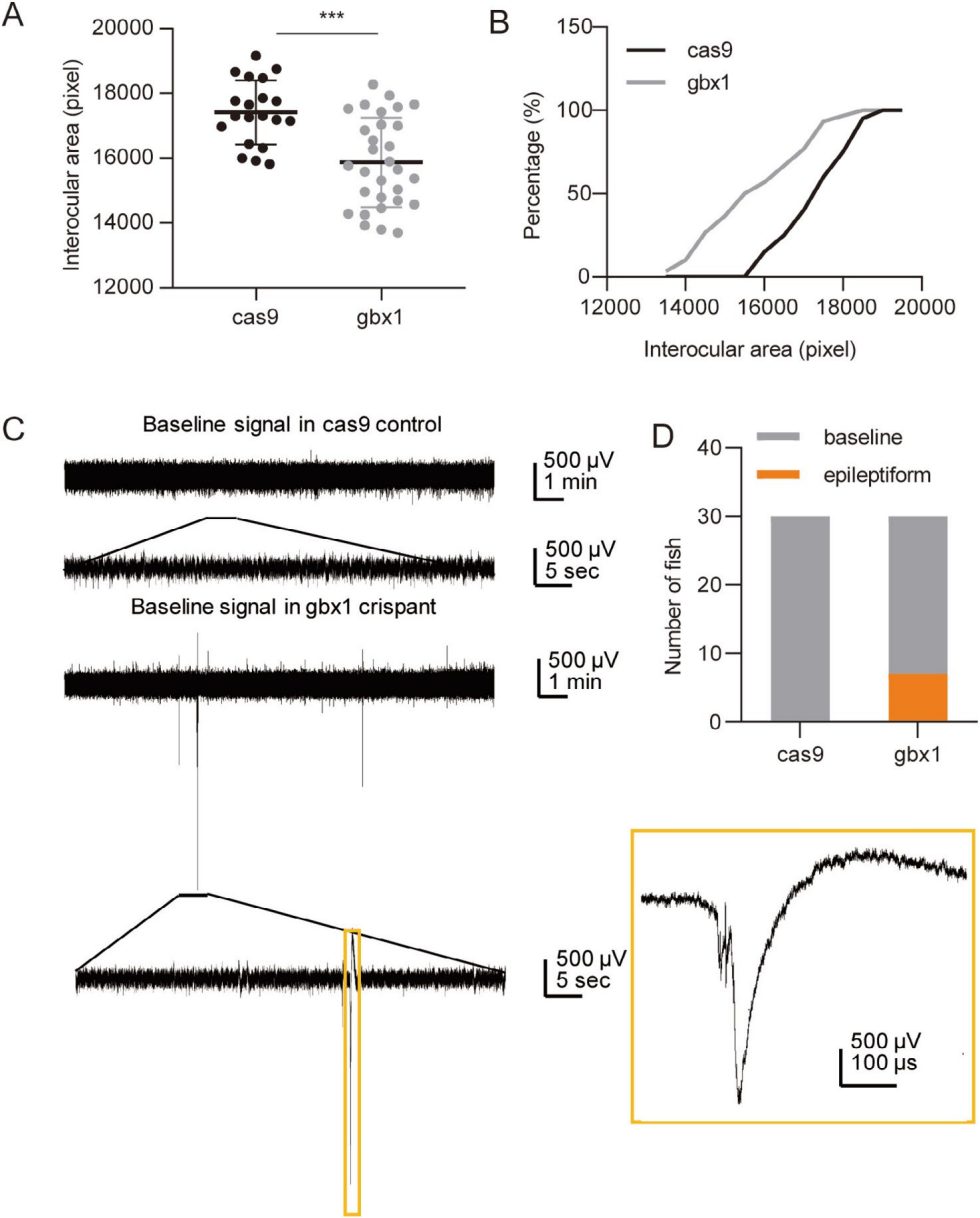
Morphology and electrophysiology of *gbx1*‐cas9 zebrafish. (A, B) The interocular area in control cas9 and *gbx1*‐cas9 groups. (C) Electrophysiological testing in two groups. (D) The distribution of zebrafish with baseline and epileptiform signals in the cas9 control group and the *gbx1*‐cas9 group. ***p < 0.001.

Representative bright‐field images of zebrafish larvae from both the Cas9 control group and the *gbx1‐*cas9 group are shown in Figure S1. The eye distance and body length are marked with blue solid lines, while the interocular area is indicated by red dashed lines (Figure S1A). A high proportion of abnormal larvae was observed, with a malformation rate of 41 out of 114 (Figure S1B). No significant difference were observed in eye distance, body length, or the eye‐to‐body length ratio (E/B ratio) between the Cas9 control group and the *gbx1*‐cas9 group. Notably, there was a significant difference in the interocular area between the Cas9 control group (*n* = 20) and the *gbx1*‐cas9 group (*n* = 30) (Unpaired *t*‐test, *p* < 0.0001) (Figure 2A,B).

In the dark environment, the spontaneous behavioral analysis of zebrafish larvae revealed no significant difference in the distance moved between the Cas9 control group and the *gbx1*‐cas9 group (Unpaired *t*‐test, *p* = 0.0581) (Figure S2A,B). Additionally, there was no significant difference in maximum speed between the two groups (Unpaired *t*‐test, *p* = 0.7702) (Figure S2C,D). The movement trajectory plots showed no noticeable differences in the density of movement trajectories or the proportion of high‐speed trajectories between the *gbx1*‐cas9 group and the Cas9 control group (Figure S2E,F).

An analysis of the 30‐s light/dark interval behaviour revealed no significant difference in swimming distance between the Cas9 control group and *gbx1*‐cas9 group during the dark phase (Unpaired *t*‐test, *p* = 0.5815) or the light phase (Unpaired *t*‐test, *p* = 0.8089) (Figure S2G). Furthermore, a non‐significant difference in maximum speed was observed between cas9/D and gbx1/D (Unpaired *t*‐test, *p* = 0.0690), as well as between cas9/L and gbx1/L (Unpaired *t*‐test, *p* = 0.9652) (Figure S2H,I).

Neurophysiological testing results revealed that in the Cas9 control group, all 30 samples exhibited normal baseline signals with no observed epileptiform activity (Figure 2C,D). In contrast, in the *gbx1*‐cas9 group, seven out of 30 samples showed epileptiform activity, while 23 out of 30 samples displayed normal baseline signals. This difference was statistically significant compared to the Cas9 control group (*p* = 0.0049).

## 4 Discussion

Here we report a patient presents with complex clinical episodes. Her physical was normal during birth, but the developmental milestones were achieved later than normal children. For 1 year old, the patient received rehabilitation therapy but the effect was not satisfactory. She has experienced episodes of abrupt awakening during sleep or napping since July 2019. The patient was diagnosed with focal epilepsy through a long‐range EEG examination. The antiepileptic drugs effectively reduced the number and severity of seizures, but they did not achieve completel remission. In addition, the MRI result was normal, indicating that the focal epilepsy had no structural brain cause.

With the increasing application of next generation sequencing, more cases of epilepsy with previously unknown causes have been found to be associated with gene variants (Chang et al. 2023). Exome sequencing covers the coding region for all the known genes. It is a powerful tool for identifying disease‐causing variants within the exome (Ross et al. 2020). Subsequent WES identified a de novo heterozygous variant in the *GBX1* gene, which may be the cause of her focal epilepsy (Figure 1D–E). It is rare and is not included in the public database. The variant is predicted to be disease‐causing through multiple software, resulting in the truncation of the protein and the loss of part of the homeodomain, which is a DNA‐binding factor involved in the transcriptional regulation of key developmental processes (Buckley et al. 2020).

To investigate the *GBX1* gene function in the nervous system, we constructed a zebrafish *gbx1* crispant model (*gbx1*‐cas9). We suggest the gbx1 function in zebrafish embryonic development through morphological, behavioral, and neurophysiological assessments. For the morphological assessments, *gbx1*‐cas9 significantly reduced the interocular area (Figure 2A,B), indicating the crucial role of gbx1 in zebrafish brain development. This finding was consistent with the previous reports (Rhinn et al. 2009; Su et al. 2014). Ectopic expression of gbx1 reduces forebrain and midbrain volume, inhibits otx2 expression, and repositions the MHB to a more anterior position. Since the *gbx1* gene regulates the development of the forebrain and midbrain, the gbx1 crispant in our study may cause structural changes in these brain areas, thereby causing the enlargement of the interocular area.

Behavioral analysis showed no significant differences in spontaneous locomotor activity or responses to light/dark transitions between the *gbx1*‐cas9 group and the Cas9 control group, suggesting that gbx1 may have little effect on zebrafish behavior in these aspects. However, neurophysiological tests showed a significant increase in epileptiform activity in the *gbx1*‐cas9 group, indicating that disruption of gbx1 affects neurological function and may lead to neurological abnormalities. This is consistent with the focal seizure phenotype observed in our patient. Interestingly, this is also related to the previously reported importance of the *Gbx1* gene in the development of the central nervous system. Loss of Gbx1 and Gbx2 in mice leads to abnormal development and increased cell death of PAX2+ interneurons in the spinal cord (Buckley et al. 2020), suggesting the important role of the *Gbx1* gene in nervous system stability. Although there are few direct reports of increased epileptic activity, the findings that *Gbx1* gene deletion results in neurodevelopmental abnormalities support our findings.

While our zebrafish *gbx1* crispant model provides valuable insights into the role of gbx1 in neurodevelopment and epilepsy, it is essential to acknowledge that animal models may not fully replicate the complexity of human neurological disorders. Zebrafish have distinct differences in brain structure and development compared to humans, which may limit the direct translation of our findings. Additionally, certain aspects of epilepsy, especially those involving higher cognitive functions, cannot be adequately assessed in zebrafish. Therefore, while our model supports the functional role of gbx1 in neurodevelopment and neurological function, further studies in mammalian models and human‐derived cell systems may be necessary to confirm these findings.

Our study has several limitations. First, the *GBX1* variant identified is located in the last exon, and NMD is unlikely to occur. Thus, the truncated protein may persist and exert a dominant‐negative or gain‐of‐function effect. While our zebrafish model supports a role for gbx1 in neurodevelopmental abnormalities, further studies are needed to elucidate the precise molecular mechanism. Second, WES cannot detect non‐coding variants, structural rearrangements, or copy number variations (CNVs), which may contribute to the patient's phenotype. Future work should include whole‐genome sequencing or array CGH to exclude these possibilities.

## Author Contributions

B.Z. designed this study, performed the experiments, and write the manuscript; X.L. performed clinical data results collection; X.Q. performed genetic testing results analysis; J.T. performed writing‐review and editing.

## Conflicts of Interest

The authors declare no conflicts of interest.

## Supporting information


**Figure S1.** Representative bright field photos of zebrafish larvae in the cas9 control group and *gbx1*‐cas9 group. (A) The blue solid line marks the eye distance and body length, and the red dotted line marks the interocular area. (B) Deformed larvae. (C–H) The eye distance, body length, and E/B ratio are in two groups.

**Figure S2.** Behavioral analysis. (A) Spontaneous behavior in a dark environment, 1 min as the time interval, light and dark stimulation behavior as 30s as the time interval. (B) The movement distance of the cas9 control group (*n* = 40) and the *gbx1*‐cas9 group (*n* = 40) were processed into a line graph, and the error bar represents the standard error [5]. (C, D) The movement distance and maximum speed of the two groups in the last 15 min were statistically analyzed. (E) 15 min trajectory diagram, the color of the motion trajectory represents the speed. (F) ≥ 40 mm/s was used as the screening threshold to form a 15‐min high‐speed motion heat map (different colors represent the frequency value).

## Data Availability

The data that support the findings of this study are available from the corresponding author upon reasonable request.
