# Supplementary figures and images for: De Novo Variant in GBX1 Gene Associated With Developmental Delay and Focal Epilepsy
Source: Mol Genet Genomic Med. 2025 Jun 16;13(6):e70114. doi: 10.1002/mgg3.70114 (PMC12168089; doi:10.1002/mgg3.70114)

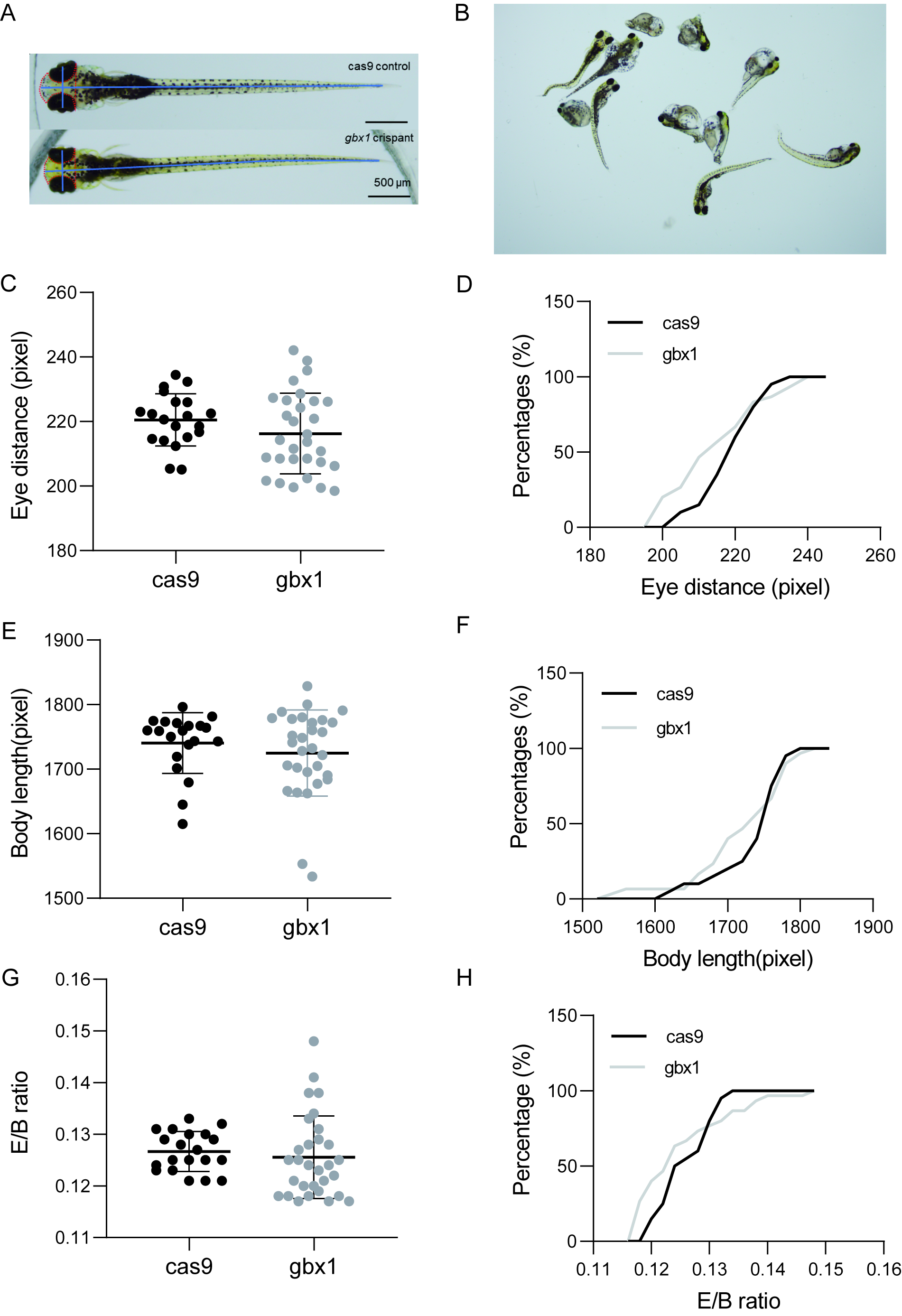

Supplement: Supplementary file 1 — Figure S1. Representative bright field photos of zebrafish larvae in the cas9 control group and gbx1‐cas9 group. (A) The blue solid line marks the eye distance and body length, and the red dotted line marks the interocular area. (B) Deformed larvae. (C–H) The eye distance, body length, and E/B ratio are in two groups. [file MGG3-13-e70114-s002.tif]

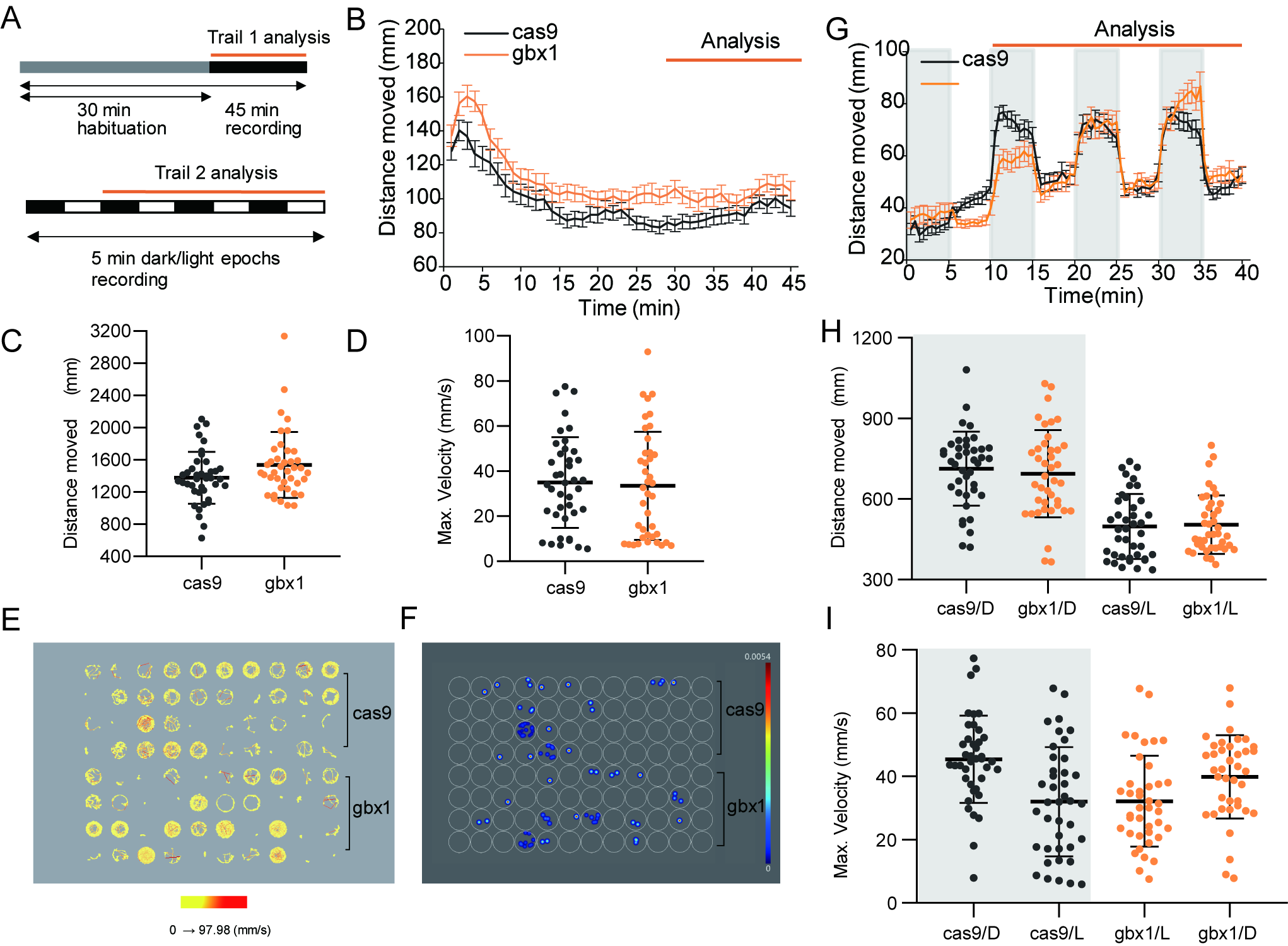

Supplement: Supplementary file 2 — Figure S2. Behavioral analysis. (A) Spontaneous behavior in a dark environment, 1 min as the time interval, light and dark stimulation behavior as 30s as the time interval. (B) The movement distance of the cas9 control group (n = 40) and the gbx1‐cas9 group (n = 40) were processed into a line graph, and the error bar represents the standard error [5]. (C, D) The movement distance and maximum speed of the two groups in the last 15 min were statistically analyzed. (E) 15 min trajectory diagram, the color of the motion trajectory represents the speed. (F) ≥ 40 mm/s was used as the screening threshold to form a 15‐min high‐speed motion heat map (different colors represent the frequency value). [file MGG3-13-e70114-s001.tif]
